# A case of severe side effects to androgen receptor inhibitor and consequently switch to radioligand therapy in early castration resistant prostate cancer

**DOI:** 10.1016/j.eucr.2024.102752

**Published:** 2024-05-11

**Authors:** Andreas Tulipan, Clemens Kratochwil, Jacob Lilleby, Wolfgang Lilleby

**Affiliations:** a Oslo University Hospital, Dept of Nuclear Medicine, Oslo, Norway; b Heidelberg University Hospital, Dept. of Nuclear Medicine, Heidelberg, Germany; c Riga Stradins University, Faculty of Medicine, Riga, Latvia; d Oslo University Hospital, Cancer Clinic-Radiumhospitalet, Oslo, Norway

**Keywords:** Prostate cancer, Androgen receptor inhibitor, Radioligand therapy

## Abstract

The development of potent novel androgen receptor inhibitors (ARi) such as apalutamide have improved the life expectancy in men with castration-resistant prostate cancer (CRPCa). However, some serious toxicity can occur limiting the choice of treatment in CRPCa. In our case, the patient experienced severe toxicity after initiation of apalutamide. Diagnostic PSMA-PET/CT confirmed the recurrence and tailored the treatment with ^177^Lu-PSMA-617 (RLT), a beta emitter radionuclide. RLT resulted in prolonged progression-free survival, thus postponing the commonly seen additional toxicity of chemotherapy.

The case highlights the possibility of early RLT in PSMA avid tumors, a treatment with minimal side-effects.

## 1 Introduction

The androgen receptor inhibitor (ARi) apalutamide and similar agents combined with ADT improve outcome in men with metastatic castration-sensitive PCa and are in general well-tolerated.^1^^,^^2^ However, unforeseen toxicity can occur to hormonal treatment and effective alternatively options to chemotherapy, the next choice of treatment, are of interest. In the last years radioligand therapy (RLT) has been approved in men with the castration-refractory stage of PCa, however, early use of RLT in men with castration-resistant disease is not established so far.^3^ In our case the novel introduction of ^177^Lu-PSMA-617 as RLT in castration resistant prostate cancer (CRPCa) tailored a less toxic approach postponing the change to chemotherapy.^4^

## 2 Case history

At the age of 64 the patient was diagnosed with locally advanced PCa, cT4N0M0, Gleason score 8, ISUP grade group 4 and initial PSA 44 ng/mL. The primary treatment with image-guided conformal radiotherapy of 78 Gy to the prostate and pelvic lymph nodes to 50 Gy was given in September 2016. He started with androgen deprivation therapy (ADT) 3 months prior to radiation and continued it. The patient had a PSA nadir of 0.28 ng/mL at the end of the radiotherapy. He was subsequently followed by his local oncologist. Due to rising PSA levels the antiandrogen bicalutamide was added in March 2020. In January 2021 PSA increased to 1,1 ng/mL and bicalutamide was discontinued and antiandrogen treatment was changed to apalutamide tablets 240 mg once daily.

**Fig. 1:**
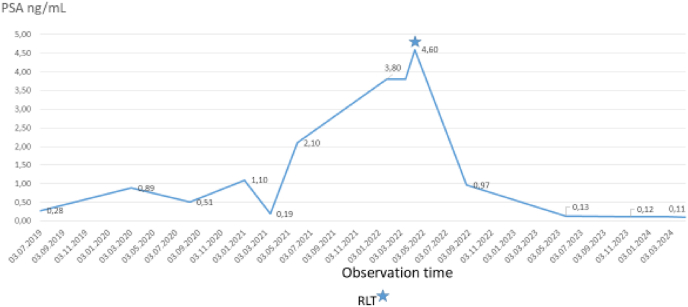
Course of serum PSA and Radioligand therapy (RLT) as indicated by asterisk*.

**Fig. 2:**
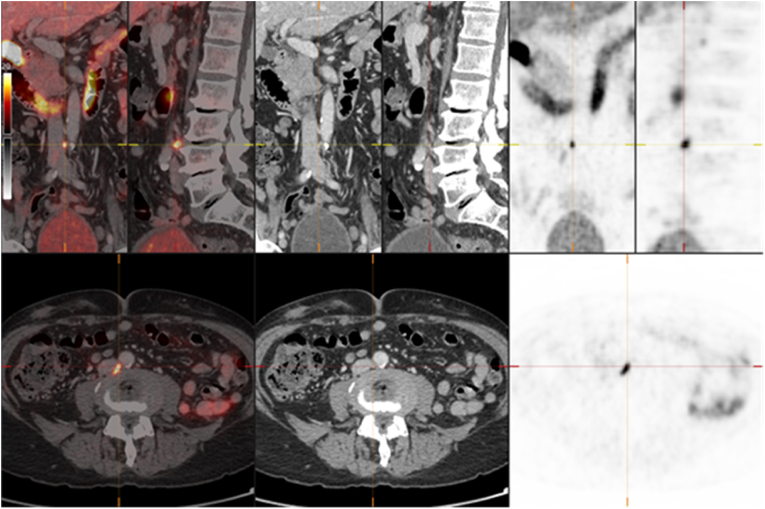
300MBq ^18^F-PSMA-1007 i. v., imaging after 120 minutes with contrast enhanced CT, Colour scale Hot Metal 0–20, PET/CT, CT and PET images in coronal, sagittal and axial plane. Metastatic lymph node marked with crosshair. (For interpretation of the references to colour in this figure legend, the reader is referred to the Web version of this article.)

Four weeks later he complained over increasing muscle cramps and myalgia and the patient developed clinical signs of kidney failure. Apalutamide was discontinued. Creatinine was measured from 109 at baseline to 177 micromol/L in February 2021. The biochemical findings disappeared gradually and the kidney function normalized. The patient recovered after 6 weeks. PSA declined to nadir 0.19 ng/mL in March 2021 (see Fig. 1). The continuous fall in PSA values confirmed the androgen dependency of the tumor. The patient was followed by watchful waiting. PSA values started to rise again in May 2021 to 2.1 ng/mL and 3.8 ng/mL in March 2022. At rising PSA level a magnetic resonance imaging was performed and revealed good treatment response to the prostate but showed no suspicious lesions in the patient. Due to the clinical discordance a PSMA-PET was accomplished (Fig. 2). The radiotracer showed high avidity in one retroperitoneal lymph node with maximum standard uptake values (SUVmax) of 28.5 and moderate avidity SUVmax of 8.8 in another lymph node detected anterior to the lumbar spine and no further signs of spread. High PSMA expression as seen in our patient is an independent biomarker of poor prognosis and dosimetry should address this. In the shared decision making the patient preferred to postpone chemotherapy and different alternative treatment options were discussed. In light of the published results from the Vision trial^3^ the patient opted for RLT with the radiopharmaceutic agent ^177^Lu-PSMA-617. In this trial patients received ^177^Lu-PSMA- 617 at a dose of 7.4 GBq. However, RLT was not available at that time in Norway. Therefore, the patient was referred to the University Clinic Heidelberg in Germany. There he received one cycle with ^177^Lu-PSMA-617 at a dose of 8.5 GBq adapted to the PSMA uptake of his lesions, a dose substantially higher compared with the dose given in the Vision trial.^5^ PSA was measured to 4.6 ng/mL at baseline. Post-RLT the PSA continued to decline to 0.11 ng/mL at last control in February 2024.

RLT was successful applied and the patient reported improved well-being, no acute xerostomia and no further long-term side-effects were observed.

## 3 Discussion

ARi in combination with ADT constitute a new treatment for men with CRPCa maintaining quality of life.^1^ After hormonal failure chemotherapy is regarded as second line treatment. However, due to rare but sometimes clinically essential side-effects, new options have to be considered. RLT has been successfully introduced in metastatic castration-refractory PCa, however, it is not known if RLT with beta emitters are beneficial to patients in earlier disease stages. In this case report severe myalgia and transient kidney function impairment stopped the option of ARi. The course of PSA suggested androgen-sensitivity in a stage of CRPCa. In search for an alternative to chemotherapy we considered RLT. RLT in PCa depends on uptake of the PSMA tracer. In our patient PSMA-PET imaging showed avid and low tumor burden. According to the back then established local protocol,^5^ a higher treatment activity than the later approved Vision dose was given. We can speculate if dose intensification has contributed to the clinical effect.

As an alternative to second line chemotherapy with its known risk to neutropenia, peripheral neuropathy, edema, hair loss and fatigue, the patient received RLT with ^177^Lu-PSMA-617. We observed a long-lasting response with RLT in a patient unfit for additive androgen blockade. Notably, after only one cycle with ^177^Lu-PSMA-617 he showed persisting suppressed PSA, good quality of life and no signs of recurrence at last FU.

## 4 Conclusion

Toxicity can be a challenge in castration-resistant PCa. Personalized treatment such as early RLT appears promising in PSMA avid low tumor burden PCa in selected patients before changing to chemotherapy.

## CRediT authorship contribution statement

**Andreas Tulipan:** Conceptualization, Validation, Writing – review & editing. **Clemens Kratochwil:** Conceptualization, Data curation, Writing – review & editing. **Jacob Lilleby:** Conceptualization, Software, Writing – review & editing. **Wolfgang Lilleby:** Conceptualization, Data curation, Writing – original draft.

## Declaration of competing interest

C. Kratochwil is co-inventor of PSMA-617 and some other PSMA-RLT related patents, presenting a potential conflict of interest related to this work.

The other authors declare to have no competing interests.
